# Item

**Published:** 1910-12

**Authors:** 


### ITEM

Battle & Co., of St. Louis, have just issued No. 14 of their series of Charts of Dislocations. This series forms a most valuable and interesting addition to any physician's library. They will be sent free of charge on application, and back numbers will also be supplied by writing Battle & Co. for them.

